# Orbital Teratoma in an Adult: A Case Report and Literature Review

**DOI:** 10.7759/cureus.98276

**Published:** 2025-12-01

**Authors:** Ryoya Nishihori, Sodai Yoshimura, Koichiro Sumi, Katsunori Shijyo, Tomonobu Kodama, Naoki Otani, Atsuo Yoshino

**Affiliations:** 1 Department of Neurological Surgery, Nihon University School of Medicine, Tokyo, JPN

**Keywords:** adult-onset orbital teratoma, diplopia, orbital tumor, proptosis, teratoma

## Abstract

Orbital teratomas are rare congenital tumors that are typically diagnosed in infancy due to rapidly progressive proptosis. Adult-onset orbital teratomas are exceedingly rare, with only a few cases reported in the literature. We present the case of a 24-year-old man who developed progressive right-sided proptosis and diplopia. Computed tomography (CT) and magnetic resonance imaging (MRI) revealed a well-circumscribed mass in the superolateral right orbit containing areas of fat attenuation without calcification or bony erosion. The tumor was completely resected via a frontotemporal craniotomy. Histopathological examination revealed mature tissues derived from all three germ layers: ectodermal (nerve bundles), mesodermal (adipose tissue and vascular elements), and endodermal (salivary gland-like glandular tissue). These findings confirmed the diagnosis of a mature teratoma. At six-month follow-up, there was no radiologic or clinical evidence of recurrence, and ocular motility had markedly improved. This case illustrates the diagnostic challenges of distinguishing adult-onset orbital teratoma from other fat-containing orbital lesions, such as dermoid cysts or lipomas. It also demonstrates that, when the lesion extends into the superolateral orbit, a frontotemporal craniotomy provides safe and complete resection with favorable functional and cosmetic outcomes.

## Introduction

Teratomas are germ cell tumors composed of tissues derived from all three embryonic germ layers: ectoderm, mesoderm, and endoderm. They most commonly occur in the gonads, sacrococcygeal region, and mediastinum. Orbital teratomas are extremely rare, and most are diagnosed shortly after birth due to significant unilateral proptosis [1].

Adult-onset orbital teratomas are exceptionally uncommon. Since the first reported case by Whitham in 1923, describing a lacrimal gland teratoma in a 21-year-old man [2], only four well-documented adult cases have been reported to date. Among the four reported adult cases, two occurred in males and two in females; all were histologically mature teratomas, typically located in the superolateral orbit. Due to their rarity, these tumors often pose diagnostic challenges and are frequently misdiagnosed as more common orbital lesions such as dermoid cysts, lacrimal gland tumors, or epidermoid cysts [3].

These tumors often remain asymptomatic for extended periods and are typically discovered only when they begin to cause symptoms due to mass effect, such as proptosis, diplopia, or visual disturbances.

In this report, we present a case of a mature orbital teratoma in a young adult male and review the relevant literature to discuss its clinical features, imaging characteristics, histopathological findings, management strategies, and prognosis.

## Case presentation

The patient was a 24-year-old man who presented with progressive right-sided proptosis, vertical diplopia on upward gaze, and decreased visual acuity in the right eye over several years. He had no history of trauma, systemic disease, or congenital anomalies, and his general health was otherwise unremarkable.

On examination, best-corrected visual acuity was 0.15 in the right eye and 1.0 in the left. Moderate proptosis was noted in the right eye, with restricted upward gaze and associated vertical diplopia. Pupillary responses were normal and symmetric, and fundoscopic examination revealed no abnormalities.

**Figure 1:**
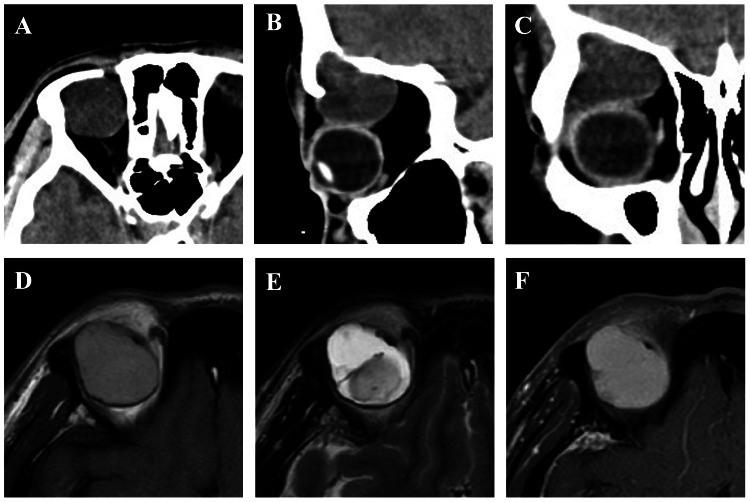
CT and MRI findings (A–C) Computed tomography (CT) scans: (A) axial, (B) sagittal, and (C) coronal sections showing a well-circumscribed 3.0-cm mass in the superolateral right orbit; (D–F) Magnetic resonance imaging (MRI): (D) T1-weighted, (E) T2-weighted, and (F) gadolinium-enhanced T1-weighted images demonstrating a hyperintense mass on both T1- and T2-weighted sequences, with minimal contrast enhancement. The lesion exhibits internal heterogeneity.

Computed tomography (CT) demonstrated a well-circumscribed, 3.0 cm mass in the superolateral right orbit. The lesion contained areas of high density in some parts, with no evidence of calcification or bony erosion (Figures 1A-1C). Magnetic resonance imaging (MRI) revealed a hyperintense mass on both T1- and T2-weighted sequences, with minimal contrast enhancement. The lesion exhibited internal heterogeneity (Figures 1D-1F).

**Figure 2:**
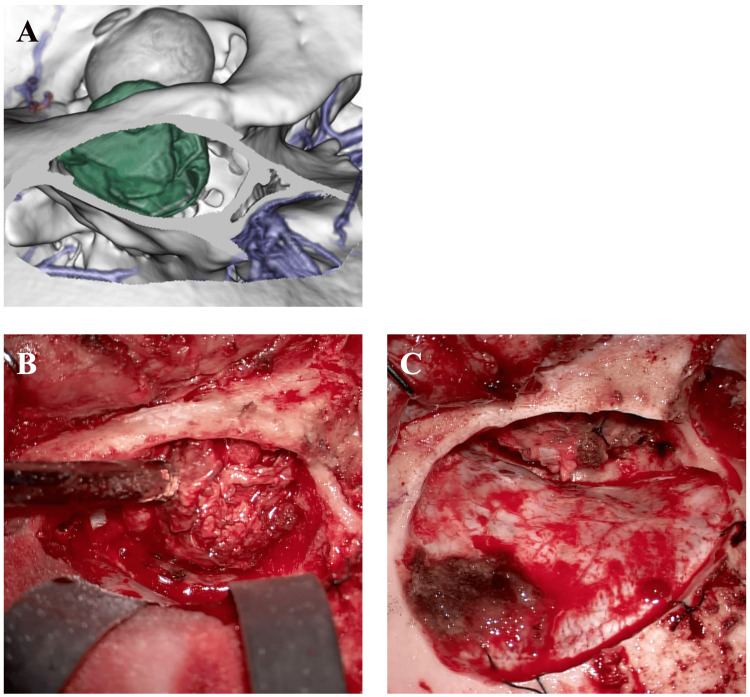
Intraoperative view (A) 3D reconstruction from computed tomography (CT) scans; (B) pre-resection view; (C) post-resection view. A right frontotemporal craniotomy was performed to access the superolateral orbit, including resection of the orbital roof.

Based on the tumor's location and imaging characteristics, differential diagnoses included dermoid cyst, teratoma, and lipoma. A right frontotemporal craniotomy with removal of the orbital roof provided direct access to the superolateral orbit. The mass was encapsulated and moderately vascular, located between the orbital periosteum and lateral rectus muscle, and could be separated from adjacent structures with relative ease. It was excised en bloc without rupture (Figures 2A-2C).

**Figure 3:**
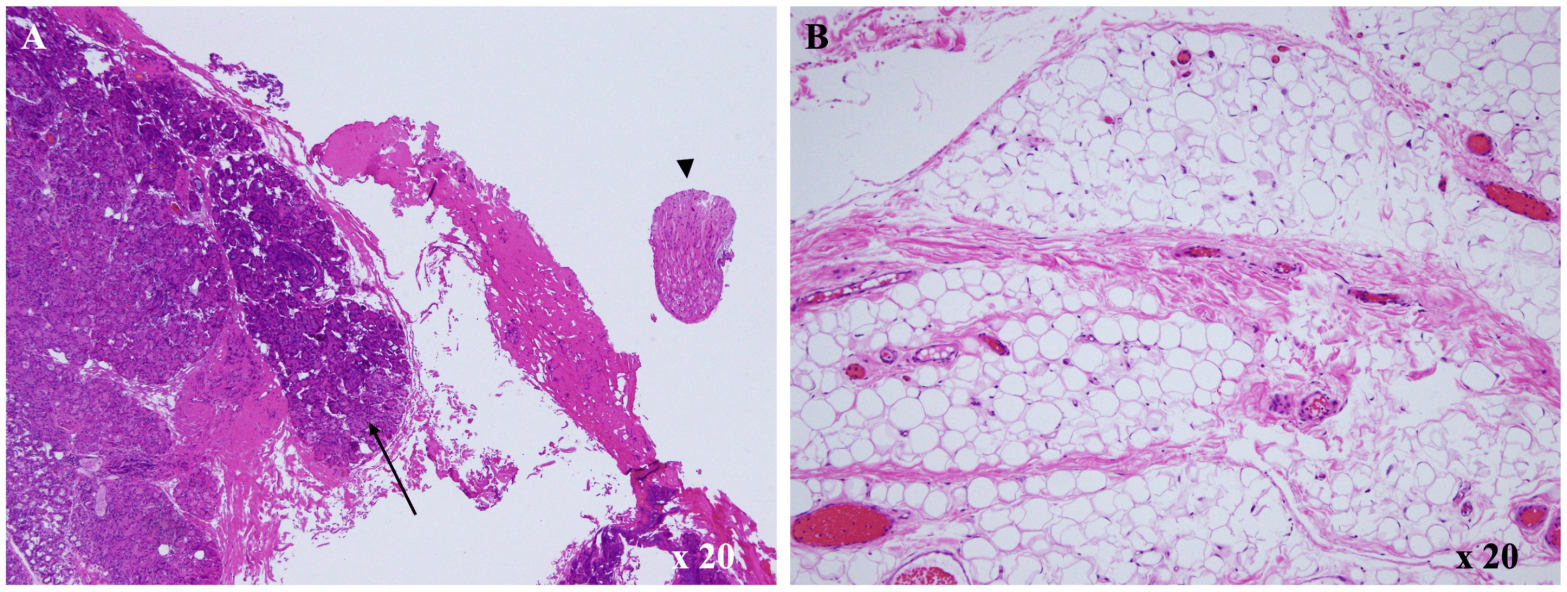
Histopathological findings Hematoxylin and eosin staining: (A) neural cords (arrowhead) and salivary gland components (arrow) were observed. (B) Adipocytes were identified.

Histopathological examination revealed mature tissues derived from all three germ layers, including salivary gland-like glandular elements (endoderm), nerve bundles (ectoderm), and adipose and vascular tissue (mesoderm). This multilayer composition distinguishes the lesion from a dermoid cyst, which contains only ectodermal derivatives. No immature or malignant components were identified, consistent with a mature teratoma (Figures 3A, 3B).

The postoperative course was uneventful. The patient experienced marked improvement in diplopia and ocular motility, with visual acuity remaining stable. Follow-up MRI at six months showed no residual or recurrent lesion. The patient continues under annual MRI surveillance.

## Discussion

Orbital teratomas are considered congenital tumors arising from totipotent germ cells that aberrantly migrate into the orbit during early embryogenesis [1,4]. While most cases are identified at birth due to marked proptosis, some lesions may remain asymptomatic for extended periods and only become clinically apparent in adulthood [2,3,5,6].

We herein summarize the previously reported patients with orbital teratoma in adults (Table 1).

**Table 1:** Summary of the previously reported cases in orbital teratoma in adults F: female; M: male; MT: mature teratoma

Case no.	Author	Year	Age	Gender	Presentation	Imaging findings	Histopathology	Surgical approach	Follow-up/Recurrence
1	Whitham [2]	1923	21	M	Gradual proptosis	Mass with embedded tooth	MT	Anterior orbitotomy	54 months/no recurrence
2	Singh et al. [5]	2013	28	F	Acute painful proptosis	Multicystic lesion with a tooth	MT	Anterior orbitotomy	Not reported
3	Rao et al. [3]	2019	20	M	Gradual orbital mass growth	Cystic lesion	MT	Lateral orbitotomy	41 months/no recurrence
4	Mukherjee & Salim [6]	2022	27	F	Gradual proptosis	Solid-cystic lesion, tooth	MT	Anterior transconjunctival approach	8 weeks/no recurrence
5	Present case	2025	24	M	Gradual proptosis	Multicystic lesion	MT	Frontotemporal craniotomy	6 months/no recurrence

The present case exemplifies this late-onset pattern. The patient had no symptoms during childhood, but as the tumor enlarged in early adulthood, he developed diplopia and limited ocular motility. Similar clinical courses have been reported by Singh et al. [5] and Mukherjee et al. [6], in which mature teratomas containing dental or glandular components were identified.

Imaging plays an important role in the diagnostic process, but is not always definitive. Fat attenuation, cystic lesions, and calcification on CT or MRI are considered characteristic features [1,4,5]. However, such features can also be observed in dermoid cysts or lipomas, and accurate diagnosis requires careful evaluation. In Singh’s report, the presence of a tooth provided a clear diagnostic clue [5], whereas in our case and that reported by Rao et al. [3], no calcification or dental elements were detected.

Histopathologically, the presence of mature elements from all three germ layers, bone, adipose tissue, glandular structures, nerves, and vasculature, is essential for the diagnosis of a teratoma. In all reported adult cases, including the present one, no immature or malignant components have been identified, and all were diagnosed as mature teratomas [2,3,5,6]. However, a case reported by Mahesh et al. [7] describing an orbital teratoma in an infant demonstrated malignant features, suggesting that the possibility of malignancy cannot be entirely excluded for orbital lesions.

Surgical excision remains the treatment of choice, and the approach depends on the tumor’s location. Anterior orbitotomy is sufficient for anteriorly located tumors, while deeper or superolateral lesions may require craniotomy. In our case, complete resection was achieved via a frontotemporal craniotomy, resulting in favorable functional and cosmetic outcomes. Similarly, Sesenna et al. reported successful removal of a huge orbitocranial teratoma in a newborn using a lateral osseous orbitotomy with temporary removal of the lateral orbital wall and partial resection of the pterion and anterior temporal bone, followed by reconstruction of the orbital floor with a bone graft and zygomatic repositioning, achieving globe preservation and good orbitofacial development [8]. These experiences support the use of tailored neurosurgical approaches for large or posteriorly extending orbital teratomas while aiming to preserve ocular function and cosmesis.

Although adult-onset orbital teratomas are rare, they may be underdiagnosed due to their resemblance to more common orbital lesions. In particular, radiologic features may mimic those of dermoid cysts or other benign fat-containing tumors, leading to misclassification. As high-resolution imaging becomes more accessible, previously unrecognized cases may be increasingly identified. Chang et al. reported that early diagnosis in a neonatal case enabled visual preservation, emphasizing the importance of early recognition [9].

Therefore, teratoma should remain in the differential diagnosis for orbital masses in adults, especially when a fat-containing, heterogeneous lesion is observed. Early surgical intervention can lead to excellent functional and aesthetic outcomes.

## Conclusions

Although orbital teratomas arising in adulthood are exceedingly rare, they should always be considered in the differential diagnosis when encountering a well-circumscribed orbital mass. Accurate diagnosis relies on careful interpretation of radiologic findings, followed by histopathological confirmation after surgical excision.

In most reported adult cases, including the present one, complete tumor resection has resulted in excellent functional and cosmetic outcomes, and the overall prognosis appears favorable. However, although no recurrence has been documented following complete excision of adult orbital teratomas, long-term imaging surveillance is recommended because of the theoretical risk of malignant transformation or delayed recurrence. This report aims to raise clinical awareness of this rare entity, adult-onset orbital teratoma, and contribute to the establishment of appropriate diagnostic and therapeutic strategies.
